# Users’ Perspectives on Primary Care and Public Health Services in the State of Rio de Janeiro, Brazil: A Cross-Sectional Study with Implications for Healthcare Quality Assessment

**DOI:** 10.3390/ijerph22091424

**Published:** 2025-09-12

**Authors:** Igor F. L. Ferraz, Mariana C. Raimundo, Natalia M. A. M. Barros, Jhoyce S. Souza, Barbará M. V. Lucio, Thiago P. Tenreiro, Edna A. Reis, Danielle Maria de Souza Serio dos Santos, Luisa A. Chaves, Brian Godman, Stephen M. Campbell, Johanna C. Meyer, Isabella Piassi D. Godói

**Affiliations:** 1 Institute of Pharmaceutical Sciences, Federal University of Rio de Janeiro, Avenida Aluízio da Silva Gomes 50—Granja dos Cavaleiros, Macaé 27930-560, RJ, Brazil; igorfradique@gmail.com (I.F.L.F.); maricrespo.r2@gmail.com (M.C.R.); natmmbarros@gmail.com (N.M.A.M.B.); jhoyce.tcc.ufrj@gmail.com (J.S.S.); bah_mvl@hotmail.com (B.M.V.L.); thiagopereira900@gmail.com (T.P.T.); dani.farma84@gmail.com (D.M.d.S.S.d.S.); luisa.arueira@gmail.com (L.A.C.); 2 Department of Statistics, Exact Sciences Institute, Federal University of Minas Gerais, Campus Pampulha, Avenida Antônio Carlos 6627, Belo Horizonte 31270-901, MG, Brazil; ednareis@gmail.com; 3 Health Technology Assessment Center—Management, Economics, Health Education and Pharmaceutical Services (GEESFAR/NATS/UFRJ) of the Federal University of Rio de Janeiro, Rio de Janeiro 27930-560, RJ, Brazil; 4 Strathclyde Institute of Pharmacy and Biomedical Sciences, University of Strathclyde, Glasgow G1 1XQ, UK; brian.godman@strath.ac.uk; 5 Department of Public Health Pharmacy and Management, School of Pharmacy, Sefako Makgatho Health Sciences University, Ga-Rankuwa, Pretoria 0208, South Africa; stephen.campbell@manchester.ac.uk (S.M.C.); hannelie.meyer@smu.ac.za (J.C.M.); 6 Antibiotic Policy Group, Institute for Infection and Immunity, City St. George’s, University of London, London SW17 0RE, UK; 7 School of Health Sciences, University of Manchester, Manchester M13 9PL, UK; 8 South African Vaccination and Immunisation Centre, Sefako Makgatho Health Sciences University, Ga-Rankuwa, Pretoria 0208, South Africa

**Keywords:** Brazil, access, quality, health services, users, public health, healthcare quality assessments

## Abstract

This study focuses on the Unified Health System (SUS) in five regions of the state of Rio de Janeiro, Brazil, one of Brazil’s most important states, as part of a comprehensive analysis of a research project, which has generated publications in earlier phases. The objective was to assess users’ perceptions of SUS in terms of access to and the quality of public health services, including primary care and pharmaceutical services. A cross-sectional study was conducted using a structured questionnaire comprising 66 questions, administered to a purposive sample of 1000 participants between August 2023 and August 2024. Data were analyzed using Pearson’s chi-square test with R software version 4.3. Among the participants, 54.5% were female, 62.5% were aged between 26 and 60 years, and 29% reported having private health insurance. Vaccination services were the most frequently used SUS service (25.1% of respondents). Participants who reported more frequent use of SUS services rated access more positively than those who used them less frequently (*p* = 0.002). The regions that evaluated SUS access and quality most favorably were Middle Paraíba and the metropolitan region, while the Coastal Lowlands region received the most negative assessments. Participants with lower socioeconomic status gave more favorable evaluations of access to public health services (*p* = 0.001). These findings highlight concerns about access to, and the quality of, SUS healthcare services and regional disparities in users’ perceptions of SUS services in Rio de Janeiro. The results underscore the importance of social participation as a key element in the evaluation and continuous improvement of responsive public healthcare.

## 1. Introduction

The 1988 Constitution marked a milestone for public health in Brazil, with Article 196 establishing health as a right for all citizens in Brazil and a duty of the State [1]. Following the institutionalization of the Unified Health System (SUS) in 1990, key principles were defined for the healthcare system. Key principles included universality, comprehensiveness, hierarchical organization, and social participation [2]. Alongside this, foundational guidelines were introduced to improve the diverse range of potential health actions [1,2]. Overall, SUS represents a significant social achievement and a substantial transformation in Brazil’s health landscape to improve the population’s well-being [1,2,3]. Managing this universal system involves financial, logistical, and human resources across different autonomous political and administrative entities (federal, state, and municipal). In this arrangement, the municipalities are responsible for providing the majority of health services [1,2]. This decentralization strategy necessitates collaboration and cooperation to align health services with local needs [4].

SUS is organized into different levels of care to enhance the rational use of available resources, which range from Primary Health Care (PHC) and Secondary Care to highly complex and specialized services in Tertiary Care facilities [5]. Within this structure, PHC plays a central role in promoting health and preventing diseases, reducing morbidity and mortality, and serving as the main entry point to health care as well as being the gatekeeper to more complex services when necessary [6]. One of the most socially recognized initiatives under SUS is the National Immunization Program (PNI). Since its inception in 1973, PNI has achieved several milestones, including the eradication of smallpox and the containment of epidemics, including COVID-19 [7].

In Brazil, the composition of primary care teams follows the guidelines of the National Primary Care Policy (PNAB). However, municipalities have the autonomy to structure their teams based on local needs, provided they meet the established minimum standards for PHC [8]. The Family Health Strategy (ESF) is the standard model, with teams comprising family physicians, nurses, community health agents (CHAs), and nursing technicians. It is also recommended that the municipalities include an Oral Health Team (ESB), primarily consisting of dentists and technicians, as well as Multidisciplinary Teams (EMulti), which include professionals such as nutritionists, physiotherapists, psychologists, and social workers [9]. Whilst the presence of a pharmacist is not mandatory in EMulti, their role is crucial in pharmaceutical services to improve the dispensing and management of medicines’ use, which directly benefits families served by PHC [10].

Whilst SUS is founded on the principle of universal and comprehensive access to services for the entire population, approximately 25% of the population has a private health insurance plan [11]. Whilst many health insurance beneficiaries have their coverage paid by their employers and not through direct payments [12], one of the main motivations for acquiring private coverage is the perceived greater ease in scheduling appointments with specialists [13]. A key reason is the current obstacles encountered in accessing SUS services, aggravated by increasing demand [14]. There can also be concerns with unequal access of funds across the different regions of Brazil to meet unmet needs, which again impacts the availability of comprehensive healthcare services [15,16,17].

Social participation is a cornerstone in ensuring that the population’s needs and demands are responded to, reflecting the foundational principles of the SUS [18]. In Brazil, Law No. 8142/1990 establishes social participation as one of the pillars of this universal system, highlighting the value of citizen engagement through mechanisms such as Health Councils and Health Conferences in shaping and strengthening the SUS [2,17]. Other countries with public health systems, including Canada [19] and the United Kingdom [20], also incorporate social participation as a key principle. The importance of public opinion in improving health systems informs SUS planning [21], underscoring the relevance of studies that explore users’ perspectives to inform future decision-making.

To collect data and foster discussions on the evaluation of various types of public health services in Brazil, the federal government developed programs such as the 2013 National Survey on the Rational Use of Medicines in Brazil (PNAUM), which aimed to evaluate, at a national level, the use and promotion of the rational use of medicines [22]. Alongside this is the 2011 Program for the Improvement of Access and Quality of Primary Care (PMAQ), which assessed the performance of health teams and the quality of services offered in primary care, aiming at the continuous improvement of care provided to the population [23]. These earlier programs contributed to surveys focusing on medication use, e.g., medication use by age group, difficulties encountered, and other findings in the country [22,23,24,25,26,27], as well as reflections on the provision of public health services [22,23,24,25,26,27]. These programs employed questionnaires as data collection tools, which subsequently served as references for the development of further research studies addressing these topics [15,16,17].

After the discontinuation of the aforementioned programs between 2015 and 2019, few publications [28,29,30] and/or studies on this topic in Brazil have been published. A systematic review was published in 2024 that focused on the challenges of universality in the SUS and evaluated access to and quality of health services in Brazil, including eight studies conducted before 2020 [28]. Additionally, a survey was published in April 2025, based on a digital questionnaire with a 4.7% response rate that included 2458 participants from various regions of the country, evaluating access to and quality of Primary Health Care. These studies showed that users who most frequently utilize the SUS belong to more socially vulnerable groups [29,30].

However, given the limited number of Brazilian publications assessing users’ perceptions of SUS services since 2020 [28,29], low response rates in previous studies [29,30], and the restriction of previous studies to specific topics, e.g., only pharmaceutical services or primary care, more up-to-date research is needed to reflect the importance of social participation in identifying population demands and needs. There is also a need to focus on a broader view of users’ perceptions of various aspects of public health across primary care, pharmaceutical services, and challenges associated with access to specialized services. In addition, the state of Rio de Janeiro—a strategic region in Brazil due to its tourism, economic, and cultural significance—faces unique challenges related to access to and quality of SUS services [31].

Consequently, a research project titled “*Assessment of Access and Quality of Public Health Services from the Perspective of the Unified Health System*” has been underway since 2023 at the Federal University of Rio de Janeiro [15,16,17]. Among the regions and the interior of the state of Rio de Janeiro. Previous phases of the project have been published, including analyses from the Coastal Lowlands [16], the metropolitan region [17], and a municipality in the Northern Fluminense region [15].

This study focuses on the five regions within the state of Rio de Janeiro: metropolitan region, Coastal Lowlands, Northern Fluminense, Mountain, and Middle Paraíba. The research aimed to engage with the population by conducting face-to-face interviews in various high-traffic locations across municipalities in both metropolitan regions and the interior of the state of Rio de Janeiro. This paper aims to analyze the perceptions of residents in these five regions regarding their access to, and the quality of, the public health system, including primary care and pharmaceutical services. Furthermore, the study aims to highlight the disparities observed among the five regions of the state of Rio de Janeiro, Brazil, associated with the provision of services in PHC, specialized care, and the pharmaceutical sector. The study also explores the main challenges faced by SUS users and their perceptions of the pharmacist’s role in health services. The findings can be used to give guidance to all key stakeholder groups in Brazil regarding potential ways to improve services in the future.

## 2. Methods

### 2.1. Study Design and Setting

**Figure 1:**
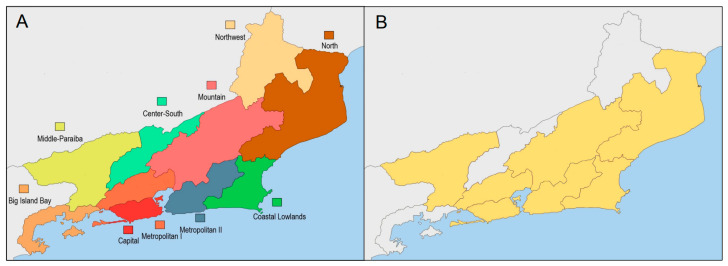
(**A**) Regions of Rio de Janeiro State in Brazil. (**B**) Regions participating in the study. Note: The regions of the state of Rio de Janeiro involved in this study are highlighted in yellow (**B**).

A cross-sectional study was conducted to evaluate the perceptions of residents from five regions of the state of Rio de Janeiro (Figure 1)—Metropolitan, Coastal Lowlands, Northern Fluminense, Mountain, and Middle Paraíba—regarding different aspects of SUS services. Because the study had no external funding and no financial resources to support the interviews, a convenience sampling was used. This took into account factors such as ease of access and travel within the municipality selected and the possibility for interviews to be conducted by undergraduate students, i.e., in municipalities where they reside and/or have family members who are part of the research project team.

According to IBGE data, the state of Rio de Janeiro has approximately 17 million inhabitants, divided into nine regions and 92 municipalities [32]. For this study, the regions were analyzed according to the political-administrative division into health regions. The metropolitan region (I and II) accounts for 75.4% of the state’s population and includes 19 municipalities: Belford Roxo, Duque de Caxias, Itaguaí, Japeri, Magé, Mesquita, Nilópolis, Nova Iguaçu, Queimados, Rio de Janeiro, São João de Meriti, Seropédica, Itaboraí, Maricá, Niterói, Rio Bonito, São Gonçalo, Silva Jardim, and Tanguá [33,34]. Among these, three municipalities were selected as a purposive convenience sample for this study: the state capital (Rio de Janeiro), São Gonçalo, and Duque de Caxias. Together, they represent 68.9% of the region’s population.

The Northern Fluminense region comprises 5.7% of the state’s population and includes 8 municipalities: Campos dos Goytacazes, Carapebus, Conceição de Macabu, Macaé, Quissamã, São Fidélis, São Francisco de Itabapoana, and São João da Barra [35]. For this study, Campos dos Goytacazes and Macaé were selected as a convenience sample, representing 52.3% of the region‘s population.

The Coastal Lowlands region accounts for 5.1% of the state’s population and includes 9 municipalities: Araruama, Armação dos Búzios, Arraial do Cabo, Cabo Frio, Casimiro de Abreu, Iguaba Grande, Rio das Ostras, São Pedro da Aldeia, and Saquarema [36]. The municipalities of Cabo Frio, Búzios, Rio das Ostras, and São Pedro da Aldeia were chosen as a convenience sample, covering 61.2% of the region‘s population.

The Middle Paraíba region represents 5.4% of the state’s population and includes 12 municipalities: Barra do Piraí, Barra Mansa, Itatiaia, Pinheiral, Piraí, Porto Real, Quatis, Resende, Rio Claro, Rio das Flores, Valença, and Volta Redonda [37]. Questionnaires were collected in all municipalities of this region.

The Mountain region accounts for 6.0% of the state’s population and includes 16 municipalities: Bom Jardim, Cachoeiras de Macacu, Cantagalo, Carmo, Cordeiro, Duas Barras, Guapimirim, Macuco, Nova Friburgo, Petrópolis, Santa Maria Madalena, São José do Vale do Rio Preto, São Sebastião do Alto, Sumidouro, Teresópolis, and Trajano de Morais [38]. In this context, Nova Friburgo, Cantagalo, and Cordeiro were included in the study, representing 21.1% of the region‘s population.

### 2.2. Data Collection Instrument

The research instrument was developed by the project team based on previous federal government projects: the National Program for Improving Access and Quality of Primary Care (PMAQ) [23,25,26,27] and the National Survey on Access, Use, and Promotion of the Rational Use of Medicines (PNAUM) [22,24] (Supplementary Material). In the development phase, the focus was on developing simple, objective, and easy-to-understand questions, following the profile of those adopted in the previously cited instruments [23,25,26,27]. The final questionnaire consisted of 66 questions, and the same instrument was applied across all five regions of the state of Rio de Janeiro included in this study. Aspects such as difficulties in the use of medicines, types of public health services utilized, and perceptions of SUS and other approaches were included.

The 66 questions were organized into the following four sections: (A) socioeconomic profile and use of health services; (B) clinical condition; (C) use of medicines; and (D) perceptions and use of public health services. It is important to emphasize that only participants who reported using SUS services answered Section D (step two: assessing users’ perceptions of access to and quality of SUS services), whereas the previous sections (A, B, and C) were completed by all participants (*n* = 1000).

Section D aimed to explore participants’ perceptions of different aspects of SUS services. It included several questions related to access to and quality of public health services, the types of services most frequently used, the need to seek health care in a municipality other than their place of residence, and other related topics. In addition, we included questions addressing the pharmaceutical context, with particular emphasis on medication use, participants’ experiences with medicine usage instructions, the acquisition and use of over-the-counter (OTC) medicines, and other relevant aspects. Additional questions sought to assess whether participants fully understood their prescriptions, including instructions for antibiotic use and polypharmacy.

To enhance the robustness of the questionnaire, it was pre-tested with 30 individuals from the municipality of Macaé (Northern Fluminense Region), near the Federal University of Rio de Janeiro (UFRJ-Macaé). During the pre-test, we confirmed that no questions required modification.

### 2.3. Data Collection and Inclusion Criteria

The sample size was calculated to estimate a proportion with a maximum margin of error of approximately 3% at a 95% confidence level, assuming the scenario of greatest variability (i.e., when the population proportion equals 0.50). This resulted in an estimated sample size of approximately *n* = 1000 participants, using the equation *n* = 1/(d^2^), distributed among the municipalities proportionally to their population sizes [39].

Because the project had no available funding, municipalities and regions of the state of Rio de Janeiro with greater accessibility and feasibility for the interview team were selected as a purposive convenience sample. Inclusion criteria required participants to be 18 years of age or older. For analyses evaluating the quality of public health services, individuals who reported never having used the SUS were excluded.

Data collection was carried out by undergraduate students from the Institute of Pharmaceutical Sciences at the Federal University of Rio de Janeiro/Macaé, who had been previously trained by the study’s coordinating professor. Interviews took place in public and freely accessible areas, generally near healthcare facilities, including municipal pharmacies, hospitals, and Basic Health Units (UBSs). These locations were intentionally selected to maximize the recruitment of participants who were users of the SUS or private health insurance, maximizing the inclusion of diverse experiences and perspectives. It should be noted that the interviewers randomly invited individuals from various high-traffic locations within each selected municipality in order to include participants with different socioeconomic profiles. The recruitment strategy aimed to approach individuals who were likely to have recent or ongoing contact with health services, particularly those present in healthcare settings at the time of data collection.

### 2.4. Data Analysis

Data analysis consisted of a descriptive assessment of participants’ responses, including questions on sample characteristics such as gender, age, education level, household income, and clinical profile, which are presented in tables and graphs. In addition, analyses were performed on utilization patterns and perceptions of public health services, with a primary focus on PHC, and included the specialized care and pharmaceutical services. To present the income profile of the interviewees, values in BRL were converted to USD using the exchange rate provided by the Central Bank of Brazil (2025), which was USD 1 = BRL 5.87 [34,40].

Additionally, users’ experiences in the pharmaceutical context were evaluated, covering the entire process of acquiring and using medicines, including over-the-counter (OTC) and prescription drugs, challenges related to polypharmacy, and patients’ perceptions of the pharmacist’s role in health care. Variables were summarized using absolute and relative frequencies, and associations between them were analyzed using Pearson’s chi-square test. Results were considered statistically significant when the *p*-value was <0.05. Analyses were conducted using Microsoft Excel 365 and R software, version 4.3.0.

In addition, we evaluated a potential association between participants’ gender, educational level, skin color, and family income and their perceptions of access to and quality of SUS services.

### 2.5. Ethical Aspects

Participation was voluntary, and research objectives were explained to participants, including a reading of the Informed Consent Form (ICF). Individuals who agreed to participate signed two copies of the ICF, one retained by the participant and the other by the researcher. Once the form was signed, the questionnaire was administered.

The present study was approved by the Ethics and Research Committee of the Federal University of Rio de Janeiro/Macaé Campus (CAAE: 68864623.6.0000.5699).

## 3. Results

### 3.1. Participant Characteristics

Based on the analysis of the 1000 questionnaires, it was observed that the sample was predominantly female (66.2%), with an average age range of 25 to 45 years (41.5%), and most participants had completed high school (33.8%).

The majority of participants reported using SUS services (92.1%), as shown in Table 1.

**Figure 2:**
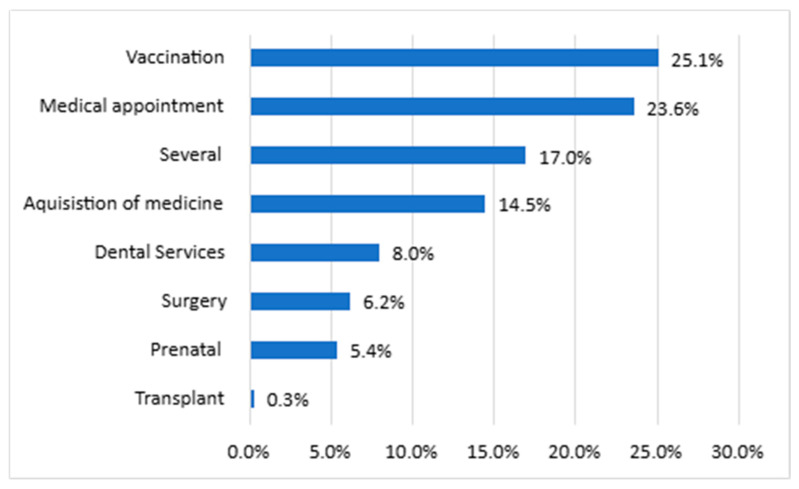
Most commonly used SUS services reported by users from the State of Rio de Janeiro (*n* = 1000). Note: Respondents were allowed to report more than one difficulty.

Among the services offered by SUS used by the participants, vaccination and medical consultations were the most frequently utilized services (25.1% and 23.6%, respectively), followed by the acquisition of medications (14.5%), as illustrated in Figure 2.

**Figure 3:**
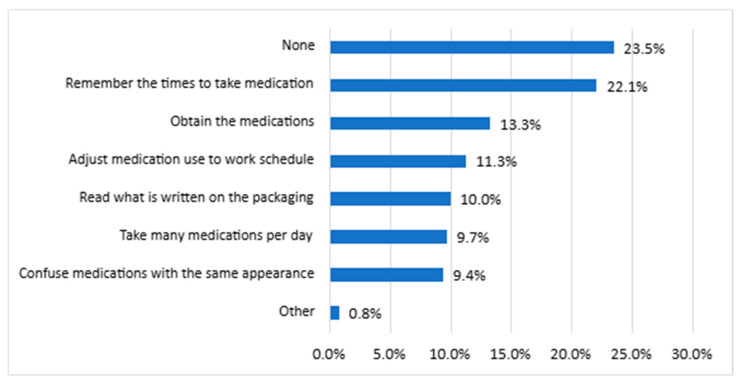
Difficulties reported by respondents associated with the use of medicines (*n* = 1000). Note: respondents could record more than one service.

25.3% of the sample reported having no difficulties in obtaining or using their medications. However, the greatest challenges identified were remembering the time to take medications (22.1%), obtaining the medication (13.3%), and adjusting medication use with their work routine (11.3%), as shown in Figure 3.

### 3.2. Responses from SUS Users

990 (99%) of the sample reported that they currently use or have previously used SUS and thus answered questions relating to the second part of the questionnaire. When asked about medical consultations, 47.4% stated that they rely solely on SUS services, 33.3% use both SUS and private health insurance, and 14.0% reported using only private health insurance for consultations. The remaining 5.3% of respondents did not answer these questions (don’t know/don’t want to answer).

Most SUS users consider it indispensable and essential, with this view shared by those who always use the system (90.8%) as well as those who use it rarely (82.1%). Among those who always use the system, 40.6% rated access to SUS services as good and 31.9% as neither good nor bad. Occasional and non-users rated access as neither good nor bad (39.0% and 47.8%, respectively). When asked about the quality of services, those who always use the system described it as very good/good, while the most negative evaluations came from those who rarely or never use the system (24.2%), as shown in Table 2.

**Figure 4:**
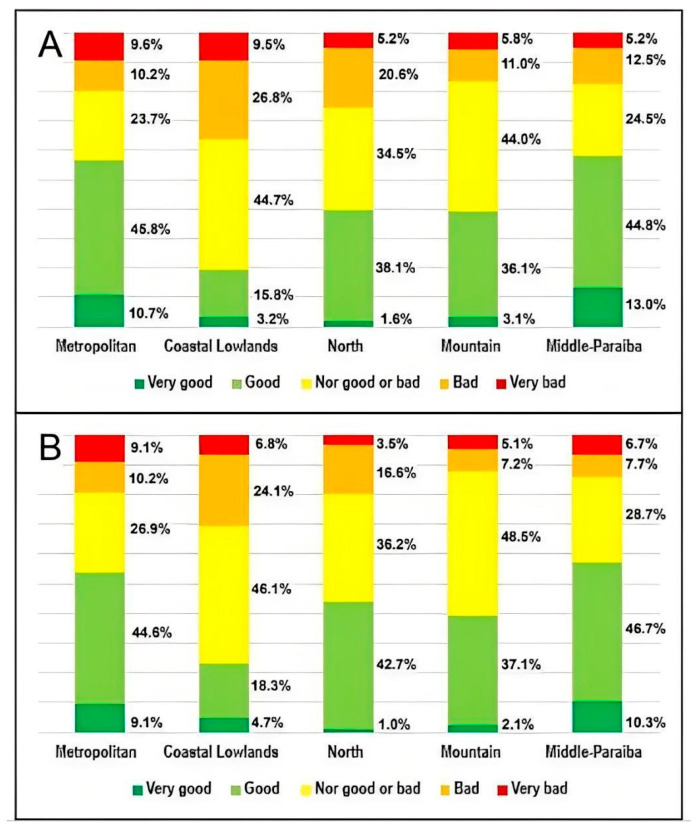
(**A**) Perception of access to SUS services in each region. (**B**) Perception of the quality of health services in each region. (*n* = 990). Notes: 11.7% of participants did not answer this question (“don’t know/don’t want to answer”); 17.3% of participants did not answer this question (“don’t know/don’t want to answer”).

Regarding perceptions of access, the Middle Paraíba and metropolitan regions received the most favorable evaluations, with 57.8% and 56.5% of respondents rating access as very good/good, respectively. In contrast, access was rated poorest (poor/very poor) in the Coastal Lowlands region, with 36.3% of users reporting negative perceptions, as shown in Figure 4. A similar pattern was observed in evaluations of service quality: the Middle Paraíba and metropolitan regions again showed the most positive responses (very good/good) from 57.0% and 53.7% of participants, respectively, while the Coastal Lowlands region had the most negative evaluations, with 30.9% rating quality as poor/very poor.

No statistically significant correlation was observed between perceptions of access to and quality of health services and factors such as gender, family income, or skin color (*p*-value > 0.05).

More favorable evaluations of access to, and quality of, health services were predominantly reported by participants with lower household incomes, with a significant association found between household income and perceptions of access to SUS services (*p* = 0.001), according to Table 3. Among respondents earning less than the minimum wage, 56.0% rated access as “very good” or “good.” Regarding service quality, 53.5% of participants in this income group rated it as “very good” or “good,” whereas 45.8% of those earning more than three minimum wages rated it as “neither good nor bad.”

Regarding how medications are obtained, 47.6% of participants reported acquiring them exclusively from private pharmacies, 40.5% obtained their medications from both private and public pharmacies, and only 9.7% reported acquiring them exclusively from public pharmacies. When asked about the role of pharmacists, the majority of those who obtained medications through the public system (either exclusively or partially) considered the pharmacist’s role essential (82.7%), while 12.3% found it indifferent. Among those who acquired medications solely through private pharmacies, 58.1% viewed the pharmacist as essential, whereas 41.9% considered their role indifferent.

Among those who acquired medications from public pharmacies only (*n* = 497), 26.8% reported never receiving guidance on how to use them, and 53.7% stated they had never received instructions on how to store their medicines. Furthermore, 40.1% said there was at least one staff member available at the pharmacy to answer questions about medications, while 41.8% said they had never encountered a pharmacist at public health units, as shown in Table 4.

## 4. Discussion

Public health services offered by SUS play a vital role for the Brazilian population, especially through primary care, which serves as the main entry point to the healthcare system. This paper shows that 93.3% of the sample reported using SUS, with those who most frequently used SUS services rating access more positively than those who used SUS less (*p*-value = 0.002). Participants with lower socioeconomic status also provided more favorable assessments of access to public health services (*p* = 0.001). Moreover, 25.1% of respondents reported using SUS for vaccination and 23.6% for medical consultations, highlighting its role in the health system.

These findings resonate with recent data from across Brazil [30], which highlights the importance of SUS in serving socially vulnerable groups and the most disadvantaged populations. This underscores the need for public policies that promote equity and address socioeconomic and geographic disparities to improve access to and quality of public health services. A high family income was the main characteristic observed in the profile of the population that reported not using SUS services, with 6.4% in this study, focusing on the state of Rio de Janeiro, and 10.7% in a national sample [29,30].

Among frequent users of SUS services, 90.8% view it as indispensable, while 82.1% of those who rarely use it share the same opinion. However, when asked about access, users who reported “always” or “frequently” using SUS expressed more favorable perceptions, with 40.6% and 44.6% rating it as “good,” respectively. These findings align with others, which showed that despite recognized issues in health services, users often express satisfaction with the available services [41,42].

When questioned about the quality of SUS services, 50.8% of frequent users rated the system as “very good,” while 44% gave a neutral evaluation (“neither good nor bad”). In contrast, among those who rarely use public healthcare services, 24.2% rated it as “poor.” These results suggest that those who use the SUS services tend to have a more positive perception compared to those who use it less frequently and may instead rely on private health plans. Similar results were seen in 2019 [41]. This is similar to the findings of the São Paulo State Department of Health, where 60.6% of hospitalized users rated the care they received as good [43]. However, 74% of those surveyed stated they faced some type of barrier when accessing healthcare services. These combined findings underscore the challenges faced by the SUS in meeting the growing demand for quality care.

In 2018, 39% of patients with chronic diseases obtained their medications from public pharmacies, with the majority of people obtaining their medicines from private pharmacies (including popular pharmacy programs) or a combination of public and private sources [44]. This study found that only 9.7% of respondents reported acquiring medications from public facilities. This disparity may be attributable to regional variations, indicating suboptimal access to medicines in public facilities within the state of Rio de Janeiro. Furthermore, given that the popular pharmacy program is administered through private pharmacies [45], respondents may have reported obtaining medications from private pharmacies even if they were procured via this public policy initiative. We will be following this up in future studies.

Among participants who obtained medications from public pharmacies in this study, the majority (82.7%) recognized the role of pharmacists as essential, even though the broader role of pharmacies is often underestimated by the public [10].

When asked about receiving guidance when obtaining medications, 26.85% of respondents stated that they had never received such guidance, and 53% reported never having received information on how to store medications or the necessary precautions. Additionally, 48.1% had never seen a pharmacist providing guidance in a UBS, Family Health Strategy (ESF), or dispensing location. A study conducted during the pandemic showed that although 95% of people perceive the pharmacist as a healthcare professional, 68.6% did not seek guidance from them, and 53.4% had never seen one [46]. This indicates an apparent disconnect between the perceived role of the pharmacist and the full utilization of their services. The lack of pharmacist presence in UBS and ESF contributes to this situation, with only 15% of UBS having a pharmacist. In the units that do have these professionals, there is better medication distribution and greater availability [46]. These findings provide guidance to community pharmacies going forward to help improve medicine use in the country and underscore the importance of continued investment in this critical health sector [47].

While 93.3% of participants reported using SUS, 31.7% reported having private health insurance, above the national average of 25%, according to the National Regulatory Agency for Private Health Insurance and Plans [48]. The higher percentage of participants who have private health insurance, compared to that expected in the country, may be associated with a better socioeconomic status and/or formal employment profile in our sample. Many Brazilians have private health insurance, based on facilities and/or benefits involving their respective companies (e.g., multinationals, state-owned companies, and service sector). Income concentration and employment opportunities are some of the incentives with which individuals seek private health coverage for their families [12]. Notably, the Coastal Lowlands and the North Fluminense regions reported the highest reliance on SUS. This is not only due to socioeconomic factors but also because the access to, and quality of, public healthcare services often influence whether users turn to private services instead [49].

The five regions in the state of Rio de Janeiro revealed varying perceptions of the health system. The Lagos region rated access and quality more negatively compared to the metropolitan region, which showed more favorable evaluations. This was also seen in the Norte Fluminense region, where a larger share of the population uses private health insurance, whereas the metropolitan region relies more heavily on the public system. Furthermore, some regions, including the Coastal Lowlands and Mountains, face shortages of UBS and ESF services. A related study conducted within this same research project in the Coastal Lowlands region [16] demonstrated that the lack of Primary Health Care units, as well as the absence of pharmacists in facilities such as UBS, has negatively impacted the population’s perception of access to and quality of health services. In addition to the findings of this study and others conducted by the same research group, it is important to note that although users’ perceptions are somewhat positive, public opinion can be significantly influenced by digital media [50]. In this context, negative aspects such as delays in care and medical errors receive greater visibility, while the system’s strengths and achievements are often downplayed or overlooked [50]. As a result, SUS has become a target of political and social attacks that threaten its structure and put at risk the continuation of one of the most important programs in the country’s history, representing a step backward in light of the progress already made in public health [50]. Consequently, it is essential to understand social demands and, above all, to develop strategies that ensure SUS is responsive to population needs and in receipt of broad social support.

Among our results, we observed that 10.5% of participants had a family income of ≥ five times the minimum wage, 60% self-identified as Black or Brown, and only 16.3% were elderly (>60 years old), according to the Brazilian classification [51]. Data from the latest demographic census of Rio de Janeiro indicate that the combined percentage of the Black and Brown population was higher than other groups across all regions of the state [32,52], with a higher proportion of women and a lower percentage of individuals over 60 [32,52], characteristics also observed in our study. Some published studies, although they did not include statistical analyses correlating respondents’ perceptions of SUS services with socioeconomic characteristics (i.e., income and education), generally observed a predominance of participants who self-identified as Black and/or brown, along with a low percentage of individuals with higher education and/or higher income [42,53,54], which is a demographic profile similar to our study.

We acknowledge that this study has some limitations, including the use of purposive convenience sampling. This strategy was adopted due to the impossibility of conducting probability sampling in a large and heterogeneous target population, composed of hundreds of thousands of SUS users. Given the lack of a unified and accessible registry of these users for random selection purposes, the questionnaire was administered to participants who were in or near health services at the time of data collection. Although this approach limits the statistical generalization of the findings to the entire SUS user population, it is frequently employed in applied health research [39,55], particularly when investigating subjective perceptions and experiences, as in the assessment of access to and quality of health services [56,57]. Furthermore, strict inclusion and exclusion criteria were applied, and efforts were made to ensure diversity in the sociodemographic profiles of participants, which we believe contributes to the internal validity of the study. In addition, considering that the study involved undergraduate students from a public university in Brazil and that the project had no available funding, the municipalities and regions of the state of Rio de Janeiro selected for participation were those with greater accessibility and feasibility in terms of transportation, accommodation, and food, allowing interviews to be conducted by a team of volunteer interviewers. Due to logistical challenges, it was not possible to include all municipalities or some localities that could have enhanced regional representation, as in the case of the mountain region, which accounts for approximately 21% of the population. Nonetheless, we prioritized areas with greater socioeconomic relevance, aiming to foster meaningful reflections on the topic within the state context. Furthermore, as a descriptive study, it has certain limitations, such as focusing on describing the findings rather than testing hypotheses or explaining mechanisms, which limits the potential for generalization [58]. However, considering the scarcity of publications on the topics addressed, coupled with the appreciable numbers of participants in this study, we believe our findings will contribute to future discussions on the regional challenges of promoting public health services within a universal system.

Overall, we believe our study offers valuable contributions to the planning and development of public health initiatives, particularly in the context of one of Brazil’s most important states as well as the other regions in Brazil facing similar challenges. Research efforts and initiatives such as projects or strategies that foster dialogue and promote the “active listening” of SUS users are essential for informed decision-making by health managers. These insights into user experiences can help guide the implementation and improvement of services to better meet the needs of the population. The results may also be of interest to other countries that face socioeconomic challenges and share the goal of expanding access to quality health services for their populations.

## 5. Conclusions

The research showed that the population of Rio de Janeiro state perceives the SUS as an essential public service, and access to and the quality of services are evaluated highly, especially by its most frequent users. It also revealed significant regional disparities within the state, particularly regarding services provided by Primary Health Care (PHC) and pharmaceutical services. Promoting research and strategies that foster continuous, constructive dialogue between the population and decision-makers is essential for developing more effective public policies tailored to local needs. To maintain the quality of health service delivery, continuous, constructive dialogue between the population and decision-makers is needed, emphasizing the importance of local engagement in ensuring both access to and the quality of services delivered by municipalities within the Brazilian federal context.

This study covered five of the seven regions of one of Brazil’s largest states. Despite being located in the Southeast, a region with more favorable socioeconomic conditions compared to the North and Northeast, user perceptions indicate that many difficulties and challenges persist, such as the provision of specialized care, especially in inland areas such as the Coastal Lowlands region. These findings reflect the real challenges faced by users and have implications for public management so that principles such as universality and comprehensiveness can be effectively achieved in practical settings.

The poor results of access to medicines in Rio de Janeiro state public facilities in comparison to national research necessitate more research to be performed to investigate this further. Finally, the need for the presence of pharmacists in community pharmacies, UBS, and ESF has been highlighted given their capacity to positively influence health indicators and the quality of life of users in alignment with the constitutional principles on which the SUS was founded.

## Figures and Tables

**Table 1:** Characteristics of study participants (*n* = 1000).

Variable	*n*	(%) *
Gender *		
Female	659	66.2%
Male	337	33.8%
Age Profile (years old)		
18–25	166	16.6%
26–45	415	41.5%
46–60	255	25.5%
More than 60	163	16.3%
Race/skin color **		
White	385	39.4%
Black	196	20.0%
Brown	390	40.0%
Other	6	0.6%
Education level ***		
Never attended school	15	1.5%
Incomplete elementary school	141	14.5%
Completed elementary school	72	7.4%
Incomplete high school	56	5.7%
Completed high school	390	40.0%
Incomplete college	145	15%
Completed college or more	156	16.0%
Family Income **** (Number of times the minimum wage *****)		
Up to 1	161	18.4%
1–2	221	26.0%
2–3	216	25.4%
3–5	264	19.3%
5–10	72	8.5%
10–20	14	1.6%
>20	3	0.4%
Use of SUS services—Yes	927	93.6%
Has a private health plan—Yes	314	31.7%

Notes: * Gender: 0.4% of participants did not answer these questions (“don’t know/don’t want to answer”); ** Race/Skin Color: 2.3% of participants did not answer these questions (“don’t know/don’t want to answer”); *** Education level: 2.5% of participants did not answer these questions (“don’t know/don’t want to answer”); **** Family income: 14.9% of participants did not answer these questions (“don’t know/don’t want to answer”); ***** Minimum wage in 2024: BRL 1412.00 (US $254.41).

**Table 2:** Perceptions of SUS users regarding the relevance, access to, and quality of the public health services (*n* = 990).

Relevance of SUS *n* (%) *						
Frequency	Indispensable		Complementary	Indifferent		*p*-Value
Always	364 (90.8%)		29 (7.2%)	8 (2.0%)		0.056
Frequently	112 (86.2%)		16 (12.3%)	2 (1.5%)		
Sometimes	219 (85.9%)		27 (10.%)	9 (3.5%)		
Rarely	156 (82.1%)		24 (12.6%)	10 (5.3%)		
ALL	851 (87.2%)		96 (9.8%)	29 (3.0%)		
Access to SUS Services *n* (%) **						
**Frequency**	**Very Good**	**Good**	**Neither Good nor Bad**	**Bad**	**Very Bad**	***p*-Value**
Always	32 (8.2%)	159 (40.6%)	125 (31.9%)	54 (13.8%)	22 (5.6%)	0.002
Frequently	6 (4.8%)	55 (44.0%)	43 (34.4%)	13 (10.4%)	8 (6.4%)	
Sometimes	10 (4.0%)	68 (27.3%)	97 (39.0%)	55 (22.1%)	19 (7.6%)	
Rarely	10 (7.6%)	48 (36.4%)	38 (28.8%)	24 (18.2%)	12 (9.1%)	
Never	1 (2.2%)	10 (21.7%)	22 (47.8%)	8 (17.4%)	5 (10.9%)	
ALL	59 (6.3%)	340 (36.0%)	325 (34.4%)	154 (16.3%)	66 (7.0%)	
Quality of SUS Services *n* (%) ***						
**Frequency**	**Very Good/Good**		**Neither Good nor Bad**	**Bad/Very Bad**		***p*-Value**
Always	196 (48.6%)		135 (33.5%)	72 (17.9%)		0.003
Frequently	66 (50.8%)		49 (37.7%)	15 (11.5%)		
Sometimes	92 (36.7%)		103 (41.0%)	56 (22.3%)		
Rarely/Never	65 (35.7%)		73 (40.1%)	44 (24.2%)		
ALL	419 (43.4%)		360 (37.3%)	187 (19.4%)		

Notes: * 1.4% of participants did not answer these questions (“don’t know/don’t want to answer”); ** 4.6% of participants did not answer these questions (“don’t know/don’t want to answer”); *** 2.4% of participants did not answer these questions (“don’t know/don’t want to answer”).

**Table 3:** Views of SUS users regarding the access to and quality of the public health services associated with family income (*n* = 990).

Access to SUS Services *n* (%)				
Family Income (Number of Times the Minimum Wage)	Very Good/Good	Nor Good nor Bad	Bad/Very Bad	*p*-Value
Up to 1	88 (56.0%)	35 (22.3%)	34 (21.7%)	0.001
1–2	80 (37.7%)	76 (35.8%)	56 (26.4%)	
2–3	85 (42.1%)	70 (34.6%)	47 (23.3%)	
3–4	53 (34.2%)	62 (40.0%)	40 (25.8%)	
>4	28 (33.3%)	39 (46.4%)	17 (20.3%)	
TOTAL *	334 (41.2%)	282 (34.8%)	194 (24.0%)	
**Quality of SUS Services *n* (%)**				
**Family Income (Number of Times the Minimum Wage)**	**Very Good/Good**	**Nor Good nor Bad**	**Bad/Very Bad**	***p*-Value**
Up to 1	85 (53.5%)	49 (30.8%)	25 (15.7%)	0.164
1–2	90 (41.5%)	80 (36.9%)	47 (21.6%)	
2–3	87 (41.0%)	84 (39.6%)	41 (19.3%)	
3–4	62 (39.5%)	63 (40.1%)	32 (20.4%)	
>4	29 (34.9%)	38 (45.8%)	16 (19.3%)	
TOTAL **	353 (42.6%)	35 (37.9%)	161 (19.5%)	

Notes: * 81.8% of participants did not answer these questions (“don’t know/don’t want to answer”); ** 55.5% of participants did not answer these questions (“don’t know/don’t want to answer”).

**Table 4:** Views of SUS users regarding acquiring medications in public pharmacies (*n* = 497).

Questions	Answers %					
	Always	Frequently	Sometimes	Rarely	Never	Total
When you pick up medications at public pharmacies of the SUS, do the staff who dispense the medications provide information and/or guidance on how to use them? *	146 (29.8%)	107 (21.9%)	67 (13.7%)	38 (7.8%)	131 (26.8%)	489 (100.0%)
When picking up medications at public pharmacies of the SUS, do you receive guidance on how to store them at home? **	68 (14.0%)	45 (9.2%)	64 (13.2%)	48 (9.9%)	261 (53.7%)	486 (100.0%)
Is the pharmacist or another staff member at public pharmacies of the SUS available when you need to ask questions about medications? ***	170 (40.1%)	87 (20.5%)	82 (19.3%)	25 (5.9%)	60 (14.2%)	424 (100.0%)
Have you ever encountered a pharmacist at the public facility you visit (ESF, UBS, or medication dispensing unit)? ****	61 (15.7%)	76 (19.6%)	57 (14.7%)	32 (8.2%)	162 (41.8%)	388 (100.0%)

Notes: * 1.6% of participants did not answer this question (“don’t know/don’t want to answer”); ** 2.2% of participants did not answer this question (“don’t know/don’t want to answer”); *** 14.7% of participants did not answer this question (“don’t know/don’t want to answer”); **** 21.9% of participants did not answer this question (“don’t know/don’t want to answer”).

## Data Availability

The original contributions presented in this study are included in the article and supplementary material. Further inquiries can be directed to the corresponding author.

## Supplementary Materials

The following supporting information can be downloaded at: https://www.mdpi.com/article/10.3390/ijerph22091424/s1, Survey Instrument.
